# Supplementary material for: Nuclear-resident RIG-I senses viral replication inducing antiviral immunity
Source: Nat Commun. 2018 Aug 10;9:3199. doi: 10.1038/s41467-018-05745-w (PMC6086882; doi:10.1038/s41467-018-05745-w)
Supplement: Supplementary file 1 — Supplementary Information [file 41467_2018_5745_MOESM1_ESM.pdf]

SUPPLEMENTARY INFORMATION

Nuclear-resident RIG-I Senses Viral Replication Inducing Antiviral Immunity

Liu *et al.*

This file includes:

Supplementary Figures 1-12

Supplementary Tables 1 and 2

Supplementary Fig. 1

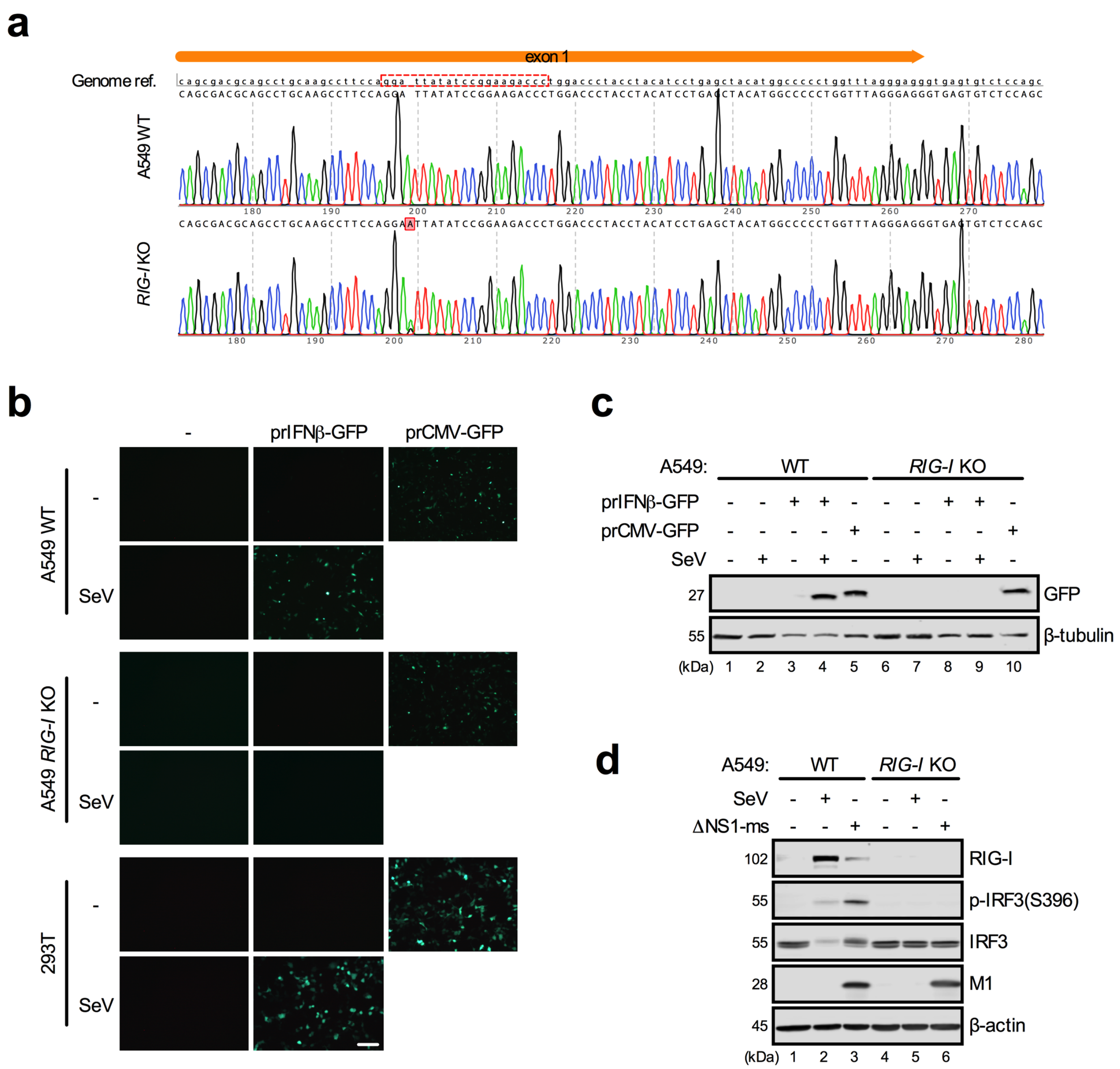

Supplementary Fig. 1. Characterization of *RIG-I* KO A549 cells (related to Fig. 1)

(a) Sequencing chromatograms of chromosome 9 genomic region flanking the sgRNA targeting site (boxed) revealed a single-nucleotide insertion into the *RIG-I* exon 1.

(b) A549 WT, *RIG-I* KO, or 293T cells were transfected with GFP expressing plasmids driven by IFNβ promoter (prIFNβ) or CMV promoter (prCMV) for 6 h followed by SeV infection (50 HAU/mL) for 16 h. GFP expression was examined and imaged by fluorescence microscopy (Leica DMI6000B). The scale bar corresponds to 50 μm. A549 *RIG-I* KO cells failed to express IFNβ promoter-driven GFP in response to SeV infection but had unaffected expression of Pol II (CMV)-driven GFP.

(c) Cell lysates from (B) were subjected to immunoblotting and probed for GFP expression.

(d) A549 WT or *RIG-I* KO cells were left uninfected, or infected with SeV (100 HAU/mL) or ΔNS1-ms (MOI = 5) for 8 h. Expression levels of RIG-I, phosphorylated IRF3, total IRF3, and M1 were examined by immunoblotting.

Supplementary Fig. 2

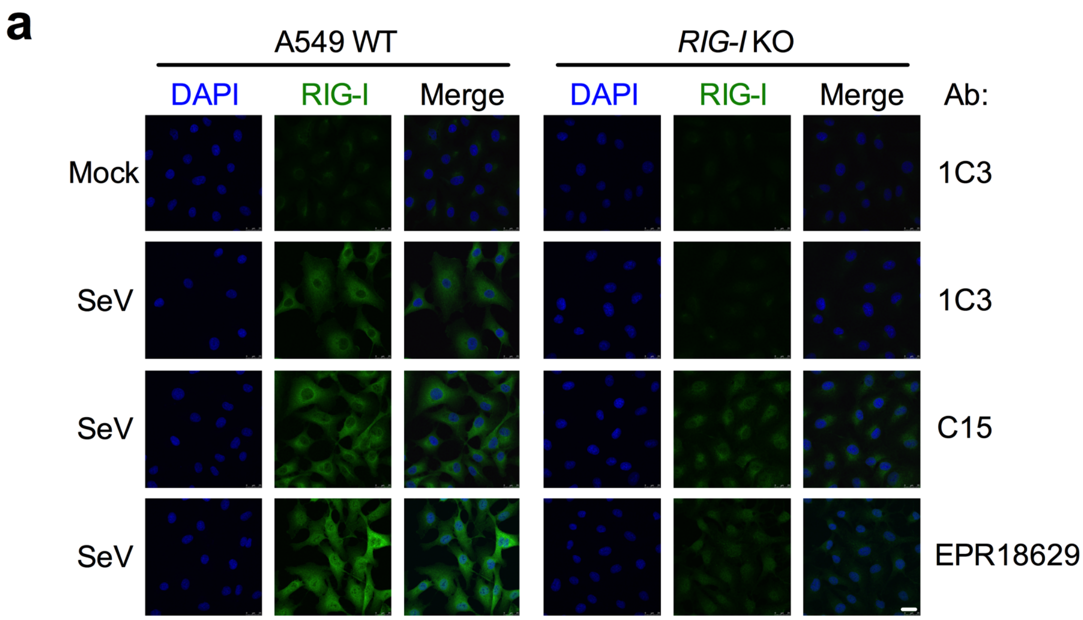

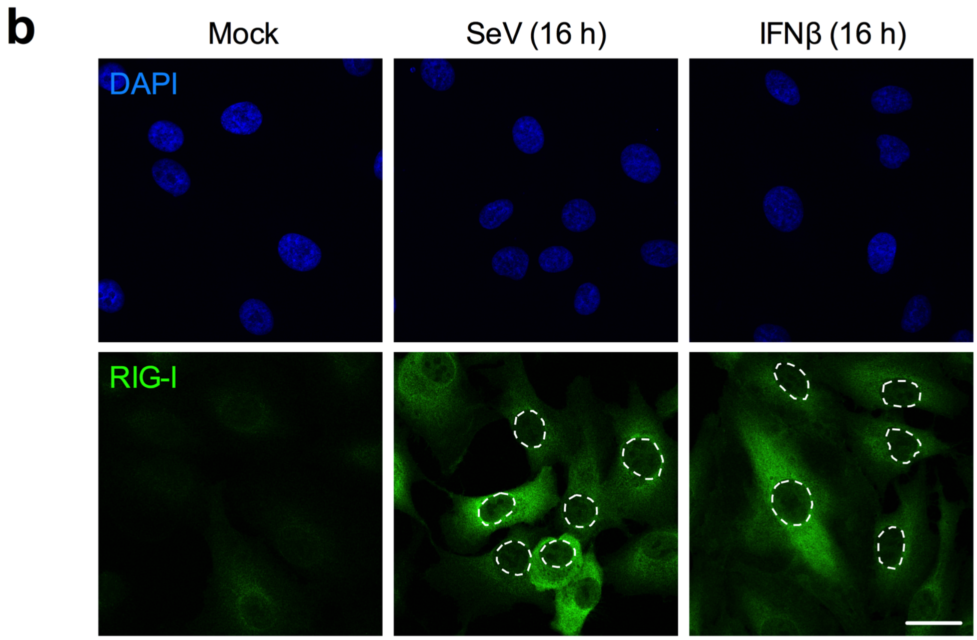

c

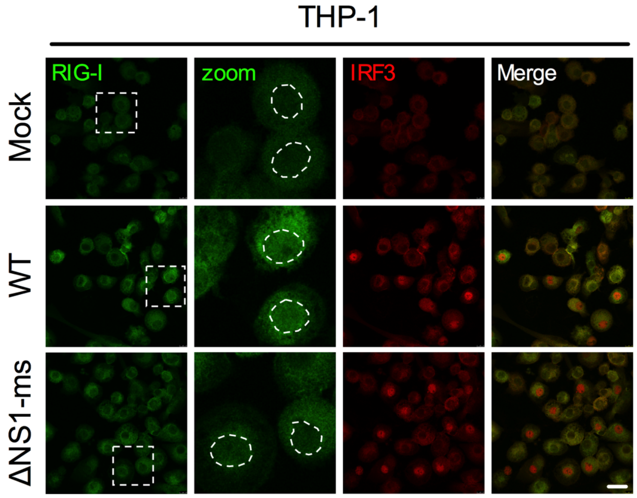
THP-1
RIG-I
zoom
IRF3
Merge
Mock
WT
ΔNS1-ms

d

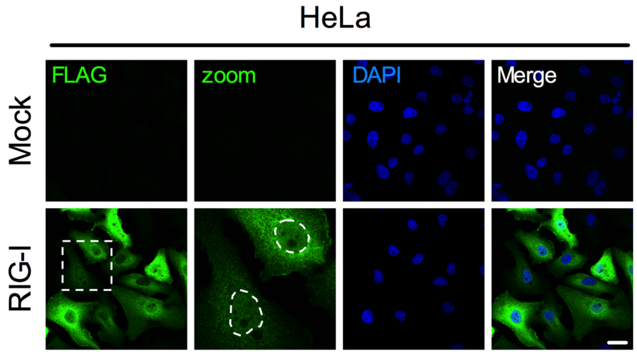
HeLa
FLAG
zoom
DAPI
Merge
Mock
RIG-I

e

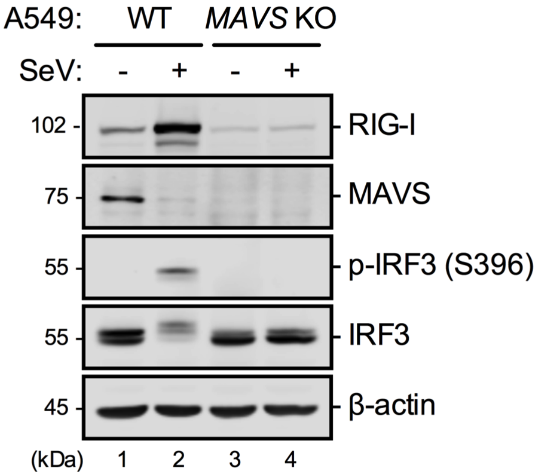
A549:
WT
MAVS KO
SeV:
-
+
-
+
102
75
55
55
45
RIG-I
MAVS
p-IRF3 (S396)
IRF3
β-actin
(kDa)
1
2
3
4

Supplementary Fig. 2. Genuine presence of RIG-I in the nucleus (related to Fig. 1)

(a) Validation of RIG-I antibodies for immunofluorescence. A549 WT or *RIG-I* KO cells were left uninfected, or infected with SeV (50 HAU/mL) for 16 h. Cells were fixed, permeabilized, and subjected to immunofluorescence with RIG-I antibodies (Ab) from various vendors, including monoclonal mouse-anti-RIG-I (1C3) (Millipore), monoclonal rabbit-anti-RIG-I (EPR18629) (Abcam), and polyclonal goat-anti-RIG-I (C15) (Santa Cruz).

(b) A549 cells were infected with SeV (50 HAU/mL) or stimulated with IFNβ (500 U/mL) for 16 h. Immunofluorescence was performed with the RIG-I antibody (1C3). The nuclear regions were outlined to highlight the nuclear RIG-I staining.

(c) THP-1 cells were differentiated with 100 nM phorbol 12-myristate 13-acetate (PMA) for 3 days before infection with WT or ΔNS1-ms PR8 virus for 8 h. Cells were subjected to immunofluorescence for RIG-I (green) and IRF3 (red).

(d) HeLa cells were transfected with FLAG-tagged RIG-I for 16 h. Cells were subjected to immunofluorescence for FLAG-RIG-I (green). Nuclei were stained with DAPI (blue). Boxed area was enlarged and the nuclear regions were outlined to highlight the nuclear RIG-I staining (c and d). The scale bar corresponds to 25 µm.

(e) Characterization of *MAVS* KO A549 cell line. A549 WT or *MAVS* KO cells were left uninfected, or infected with SeV (50 HAU/mL) for 8 h. Expression levels of RIG-I, MAVS, phosphorylated IRF3, and total IRF3 were monitored by immunoblotting.

Supplementary Fig. 3

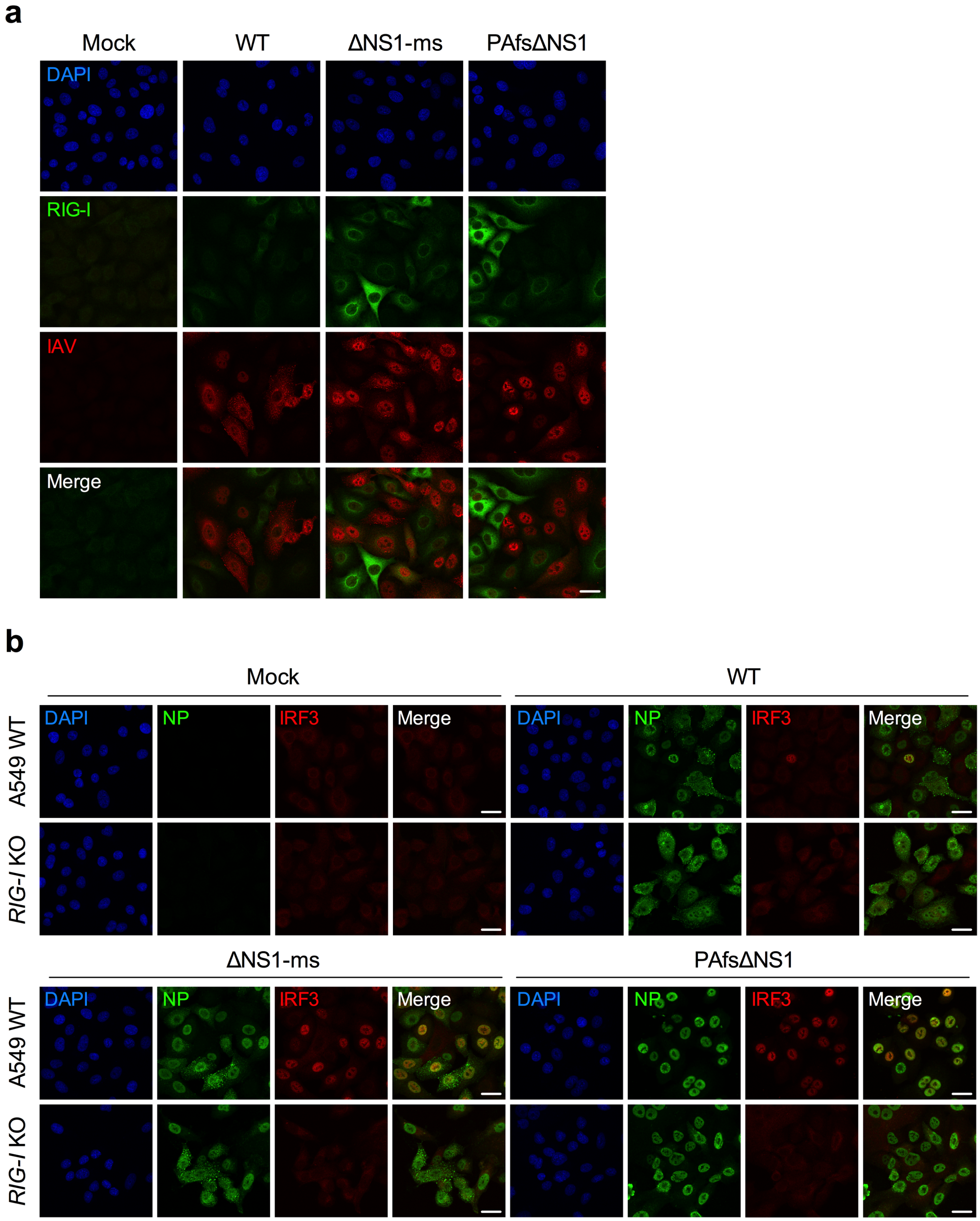

Supplementary Fig. 3. RIG-I dependent IAV sensing upregulates RIG-I expression in neighboring cells (related to Fig. 2)

(a) A549 cells were left uninfected, or infected with WT, ΔNS1-ms, or PAfsΔNS1 PR8 virus (MOI = 2) for 14 h. Cells were subjected to immunofluorescence for RIG-I (green) and IAV (red). Nuclei were stained with DAPI (blue).

(b) WT or *RIG-I* KO A549 cells were left uninfected, or infected with WT and the panel of mutant PR8 viruses (MOI = 10) for 8 h. Immunofluorescence was performed to determine the localization of viral NP protein (green) and IRF3 nuclear translocation (red). Nuclei were stained with DAPI (blue). The scale bar corresponds to 25 μm.

Supplementary Fig. 4

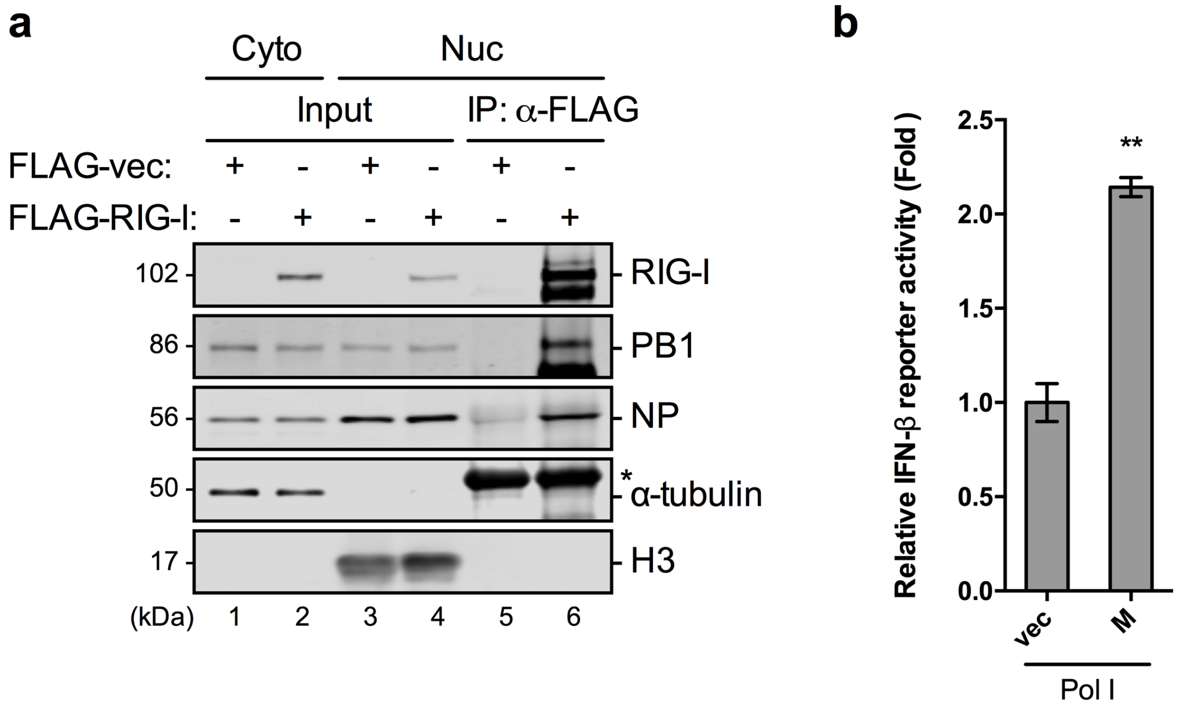

Supplementary Fig. 4. Nuclear RIG-I associates with vRNP of IAV in RNP reconstitution (related to Fig. 3)

(a) 293T cells were RNP reconstituted with the M segment in the presence of FLAG vector or FLAG-RIG-I for 24 h. Cells were subjected to cellular fractionation and the nuclear fractions were immunoprecipitated (IP) with the FLAG antibody. Expression levels of FLAG-RIG-I, PB1, and NP were determined by immunoblotting. α-tubulin and histone H3 served as the markers for the cytoplasmic and nuclear fractions, respectively. The heavy chains of IP antibodies are indicated by an asterisk (*).

(b) 293T cells were RNP reconstituted with the Pol I vector or M segment in the presence of FLAG-RIG-I. FLAG immunoprecipitates from the nuclear fractions were extracted with TRIzol Reagent (Invitrogen) and the associated RNA (50 ng) was tested for immunostimulatory activity in 293T cells transfected with p125Luc and pTK-rLuc. RLUs were determined at 24 h after RNA transfection and expressed as fold change relative to the Pol I vector control. Data are shown as mean ± SD of two independent experiments. Significant difference was determined by an unpaired Student's *t* test. ***p* < 0.01.

Supplementary Fig. 5

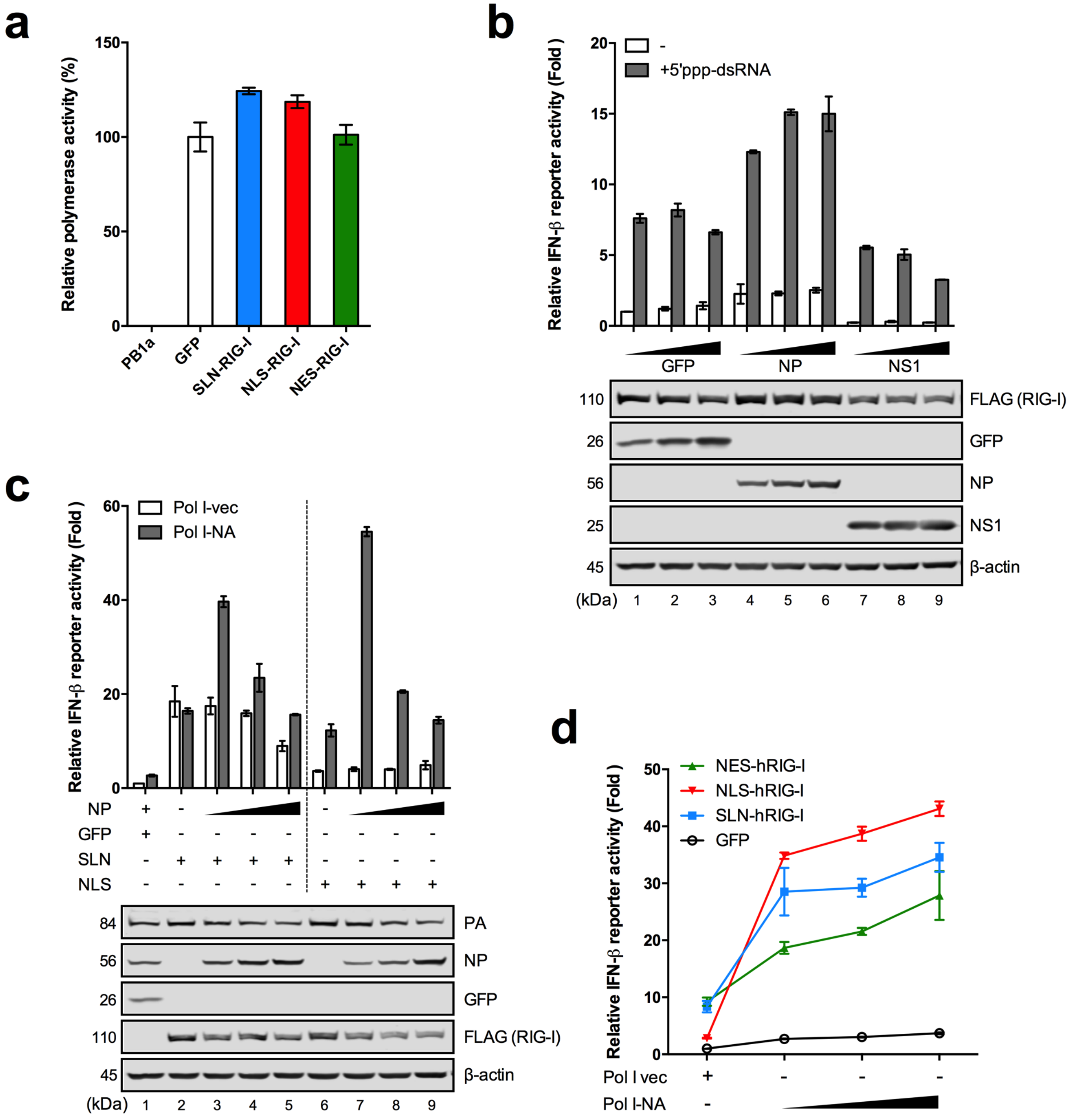

Supplementary Fig. 5. Levels of NP expression affect the generation of immunostimulatory RNA during RNP reconstitution (related to Fig. 3)

(a) 293T cells were cotransfected with four Pol II-driven plasmids (50 ng each) expressing viral polymerase subunits (PB2, PB1, and PA) and NP, a Pol I-NP-fLuc plasmid expressing reporter vRNA (50 ng), pTK-rLuc (10 ng), along with plasmids encoding GFP, SLN-, NES-, or NLS-RIG-I (100 ng). Reconstitution in the presence of a catalytically inactive PB1 subunit (PB1a) served as a negative control. RLUs were determined at 24 h.p.t and expressed as percentage changes compared to the GFP group.

(b) 293T cells were cotransfected with pCMV-FLAG-RIG-I (50 ng), p125Luc (100 ng), pTK-rLuc (10 ng), and increasing amount of Pol II-driven GFP, NP, or NS1 (50, 100, and 200 ng) for 6 h followed by stimulation with the 19-bp 5'ppp-dsRNA (100 ng) for 18 h. RLUs were expressed as fold change relative to the GFP (50 ng) control without RNA stimulation. Cell lysates were subjected to immunoblotting for FLAG-RIG-I, GFP, NP, and NS1 expression.

(c) 293T cells were RNP reconstituted with Pol I vector or PR8 NA segment (50 ng) in the absence or presence of increasing amount of pcDNA-NP (50, 100, and 200 ng) along with pCMV-FLAG-SLN- or NLS-RIG-I (100 ng), p125Luc (100 ng), and pTK-rLuc (10 ng). RLUs were measured at 24 h.p.t and expressed as fold change relative to the Pol I vector reconstitution in the presence of 50 ng pcDNA-NP and 100 ng pcDNA-GFP (lane 1). Cell lysates were subjected to immunoblotting for FLAG-RIG-I, GFP, NP, and PA expression.

(d) 293T cells were RNP reconstituted with Pol I vector (50 ng) or increasing amount of Pol I-NA (50, 100, and 200 ng) along with plasmids encoding GFP, SLN-, NES- or NLS-RIG-I (100 ng), p125Luc (100 ng), and pTK-rLuc (10 ng). RLUs were measured at 24 h.p.t. and expressed as fold change relative to the Pol I vector reconstitution in the presence of GFP. Data are shown as mean ± SD of three independent experiments performed in triplicates.

Supplementary Fig. 6

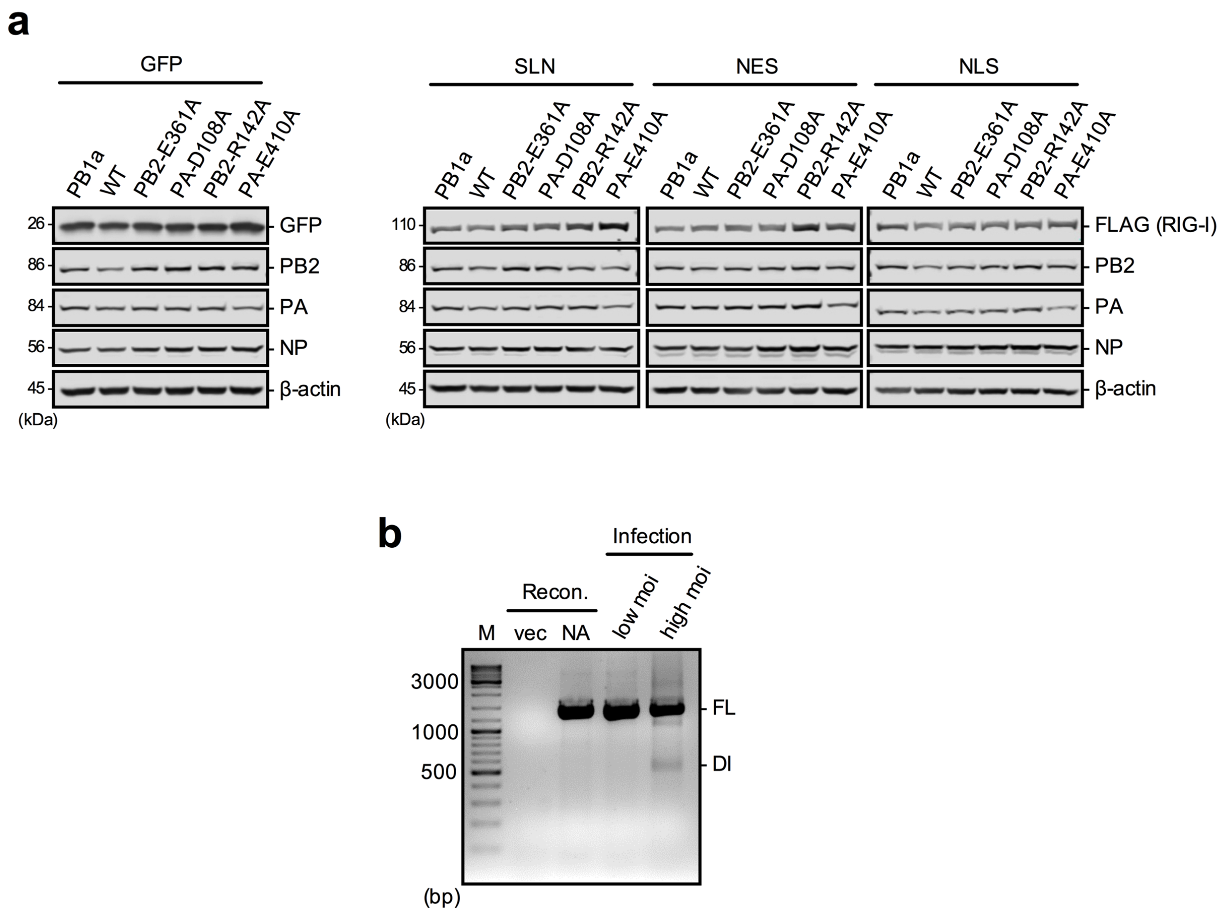

Supplementary Fig. 6. Expression levels of mutant IAV polymerases and DI genome detection in RNP reconstitution (related to Fig. 3)

(a) Expression levels of GFP, FLAG-RIG-I, and mutant IAV polymerases in Fig. 3f were determined by immunoblotting.

(b) Total RNA was extracted from 293T cells RNP reconstituted with Pol I vector or PR8 NA segment using TRIzol and subjected to RT-PCR using NA segment-specific primers located in the 3' and 5' non-coding regions (NCR) to detect the full-length (FL) and defective-interfering (DI) RNA simultaneously. Viral genomic RNA extracted from PR8-infected culture supernatants after passages at a low MOI (0.001) and a high MOI (10 for three consecutive passages) was amplified and served as controls for the absence and presence of DI RNA derived from the NA segment, respectively.

Supplementary Fig. 7

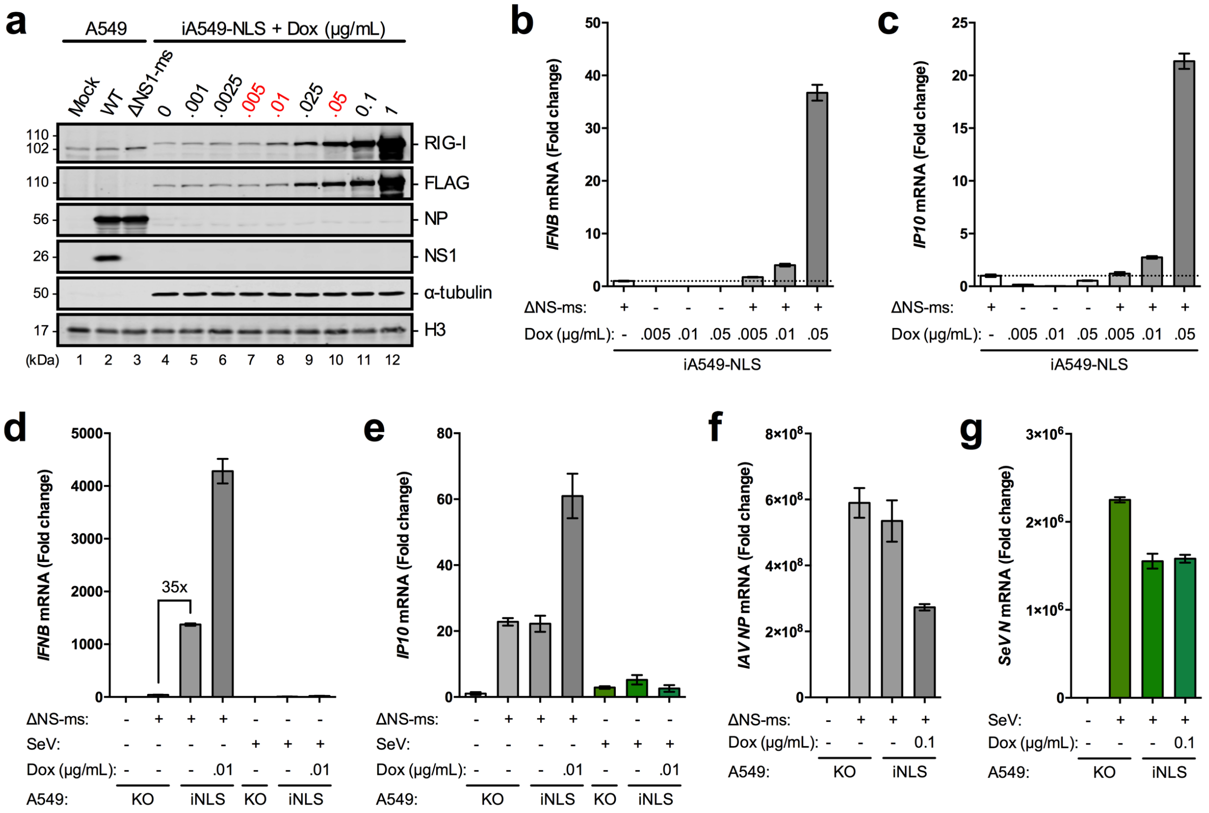

Supplementary Fig. 7. Physiologically comparable levels of nuclear RIG-I are sufficient for sensing IAV, but not SeV replication (related to Fig. 4 and 6)

(a) A549 *RIG-I* KO cells expressing NLS-RIG-I were induced with a range of Dox concentration (0 to 1 μg/mL) for 4 h and the expression levels of NLS-RIG-I in the whole cell extracts were compared with that of the endogenous nuclear RIG-I in the nuclear fractions of wild-type A549 cells left non-infected or infected with WT PR8 or ΔNS1-ms (MOI = 5) for 6 h. Immunoblotting was performed to detect the levels of viral NP and NS1 proteins. RIG-I expression was probed by both RIG-I and FLAG antibodies, with the latter specifically detecting NLS-RIG-I which contains a tandem FLAG tag. α-tubulin and histone H3 served as the markers for the cytoplasmic and nuclear fractions, respectively. Three doses of Dox (marked in red) were selected for subsequent NLS-RIG-I induction to achieve comparable or slightly higher levels than the endogenous nuclear RIG-I.

(b and c) A549 *RIG-I* KO cells expressing NLS-RIG-I were left non-induced or induced with the selected doses of Dox for 4 h, followed by mock or ΔNS1-ms infection (MOI = 1) for 14 h. The mRNA levels of IFNβ (b) and IP10 (c) were determined by qRT-PCR. Relative mRNA expression was normalized to GAPDH mRNA levels and expressed using the ΔΔCt method relative to the condition with infection but without Dox induction (Column 1).

(d-g) A549 *RIG-I* KO cells expressing NLS-RIG-I were left non-induced or induced with 0.01 μg/mL Dox for 4 h, followed by infection with ΔNS1-ms (MOI = 1) or SeV (50 HAU) for 14 h. In parallel, *RIG-I* KO cells were left uninfected or infected under the same conditions. The mRNA levels of IFNβ (d), IP10 (e), IAV NP (f), and SeV N (g) were determined by qRT-PCR. Relative mRNA expression was normalized to GAPDH mRNA levels and expressed using the ΔΔCt method relative to the uninfected *RIG-I* KO cells (Column 1). Data are shown as mean ± SD of two independent experiments.

Supplementary Fig. 8

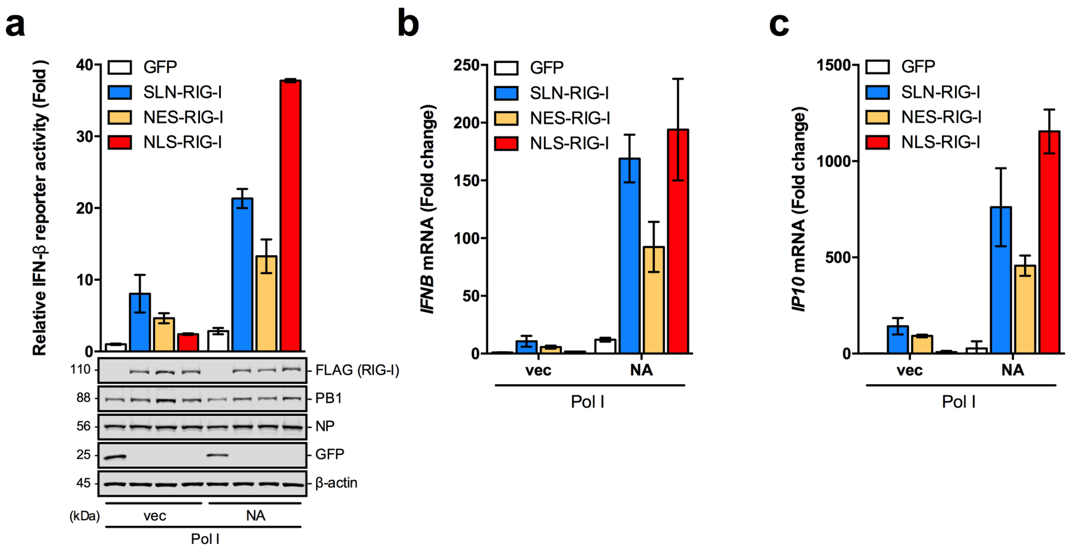

Supplementary Fig. 8. IFNβ promoter activation by compartment-specific RIG-I as determined by luciferase reporter assay correlates with antiviral gene expression (related to Fig. 3 and 4)

(a) 293T cells were RNP reconstituted with Pol I vector or PR8 NA segment in the presence of GFP, SLN-, NES-, or NLS-RIG-I for 24 h. RLUs were expressed as fold change relative to the Pol I vector reconstitution in the presence of GFP. Expression levels of FLAG-RIG-I, GFP, PB1, and NP were determined by immunoblotting.

(b and c) 293T cells were RNP reconstituted as in (a) and the mRNA levels of IFNβ (b) and IP10 (c) were determined by qRT-PCR. Relative mRNA expression was normalized to GAPDH mRNA levels and expressed using the ΔΔCt method relative to the Pol I vector reconstitution in the presence of GFP. Data are shown as mean ± SD of two independent experiments.

Supplementary Fig. 9

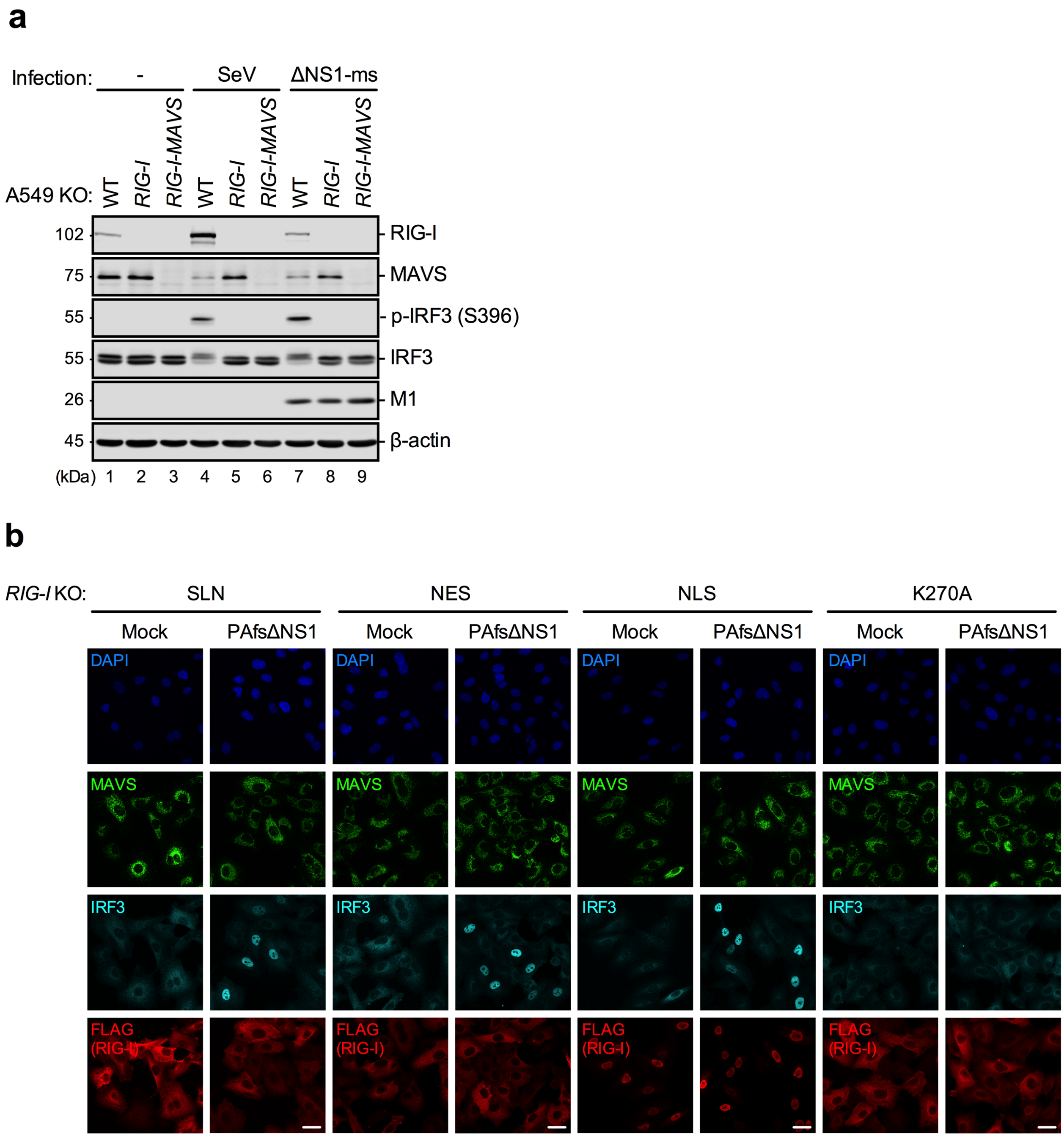

Supplementary Fig. 9. MAVS localization upon IAV infection in the presence of compartment-specific RIG-I (related to Fig. 4)

(a) Characterization of *RIG-I-MAVS* DKO A549 cell line. A549 WT, *RIG-I* KO, or *RIG-I-MAVS* DKO cells were left uninfected, or infected with SeV (50 HAU/mL) or ΔNS1-ms (MOI = 5) for 8 h. Expression levels of RIG-I, MAVS, phosphorylated IRF3, total IRF3, and M1 were monitored by immunoblotting.

(b) *RIG-I* KO A549 cells inducibly expressing SLN-, NES-, NLS-, or K270A-RIG-I were induced with 1 µg/mL Dox for 4 h followed by infection with PAfsΔNS1 virus (MOI =10) for 6 h. Cells were subsequently subjected to immunofluorescence for MAVS (green), IRF3 (cyan), and FLAG-RIG-I (red). Nuclei were stained with DAPI (blue). The scale bar corresponds to 25 µm.

Supplementary Fig. 10

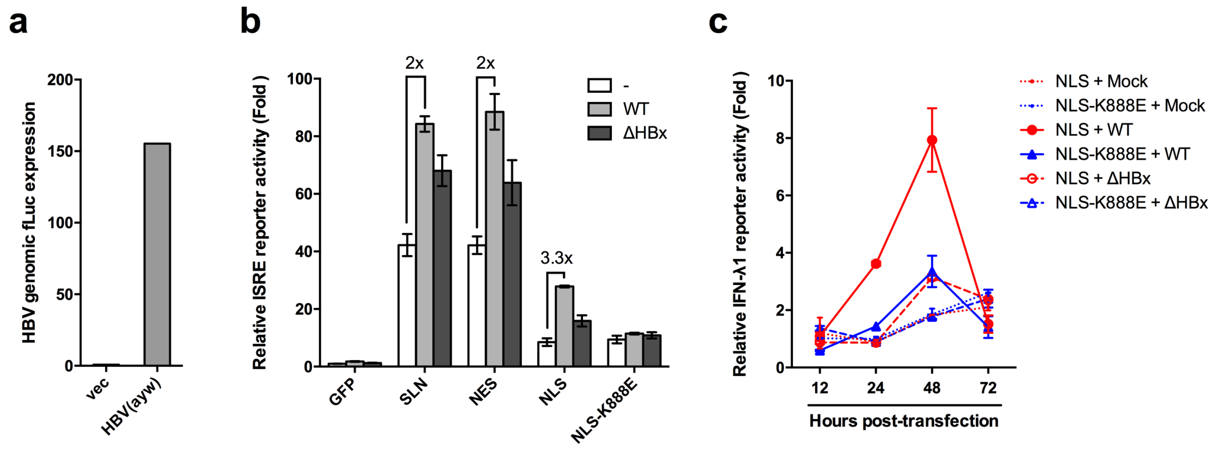

Supplementary Fig. 10. Sensing of HBV pgRNA by NLS-RIG-I requires its RNA binding activity (related to Fig. 6)

(a) Characterization of the Huh-7 cells stably transfected with an empty vector or a greater-than-unit-length HBV genome (pawy1.2) carrying a firefly luciferase reporter (fLuc).

(b) 293T cells were cotransfected with WT (pawy1.2) or ΔHBx (pawy1.2*7) HBV genome (200 ng) along with plasmids encoding GFP, SLN-, NES-, NLS-, or NLS-K888E-RIG-I (100 ng), pGL-3xISRE-fLuc (100 ng), and pTK-rLuc (10 ng). RLUs were measured at 48 h.p.t. and expressed as fold change relative to the mock transfection in the presence of GFP.

(c) 293T cells were cotransfected with WT (pawy1.2) or ΔHBx (pawy1.2*7) HBV genome (200 ng) along with plasmids encoding NLS- or NLS-K888E-RIG-I (100 ng), pGL-IFNλ1-fLuc (100 ng), and pTK-rLuc (10 ng). RLUs were measured at indicated time points post-transfection and expressed as fold change relative to the mock transfection in the presence of NLS-K888E-RIG-I at 12 h. Data are shown as mean ± SD of two independent experiments performed in triplicates (b, c).

Supplementary Fig. 11

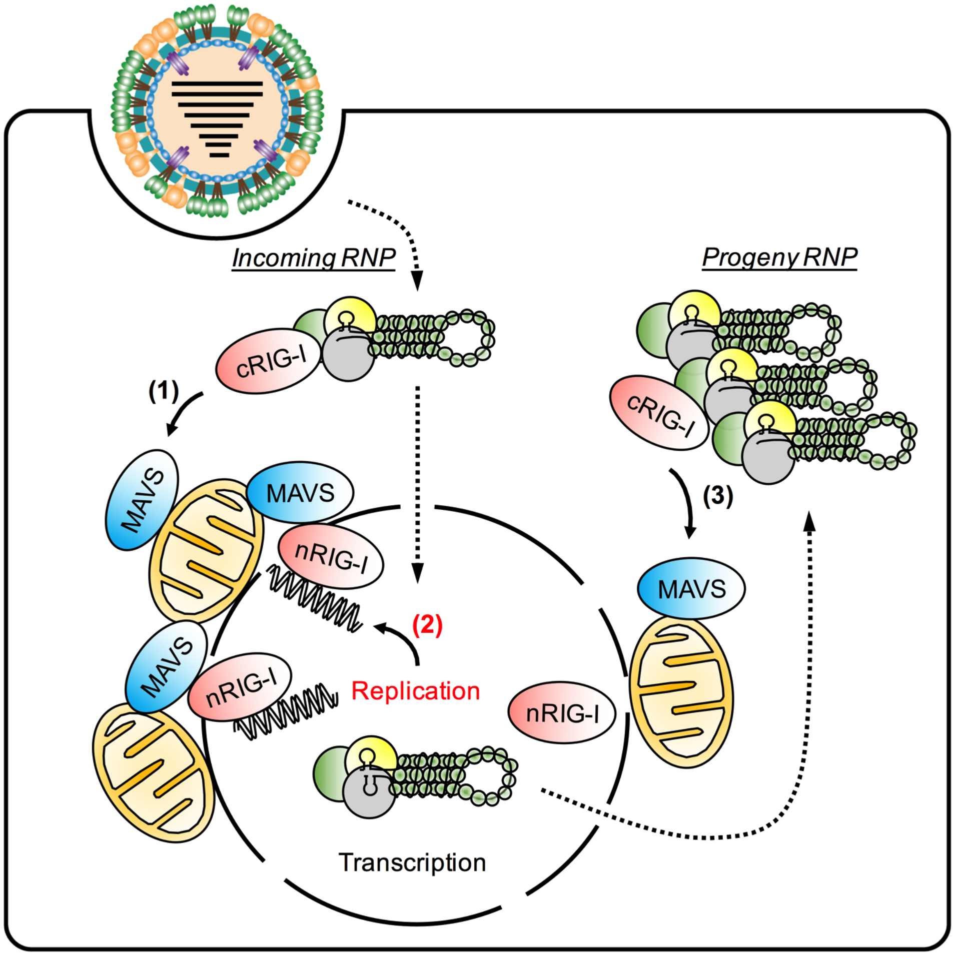

Supplementary Fig. 11. Proposed model for the formation of a perinuclear depot for nuclear RIG-I sensing of IAV replication

(1) Limited incoming vRNPs are sensed by cytoplasmic RIG-I (cRIG-I); (2) with the onset of nuclear viral replication, virus-derived PAMPs are captured by nuclear RIG-I (nRIG-I) and such signaling-competent complexes relay antiviral signals to MAVS located on perinuclear mitochondria once the nuclear membrane architecture (nuclear pores) is compromised; (3) progeny vRNPs following nuclear export provide additional PAMPs for cRIG-I sensing.

Supplementary Fig. 12. Uncropped blots

Figure 1b

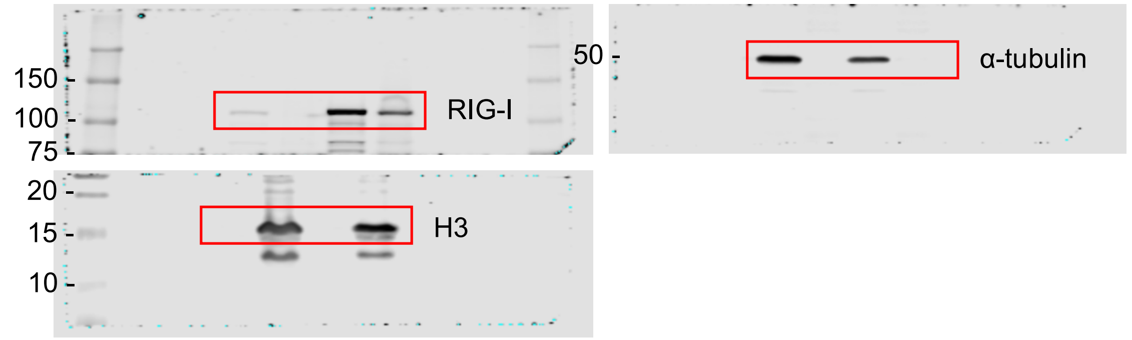

Figure 1c

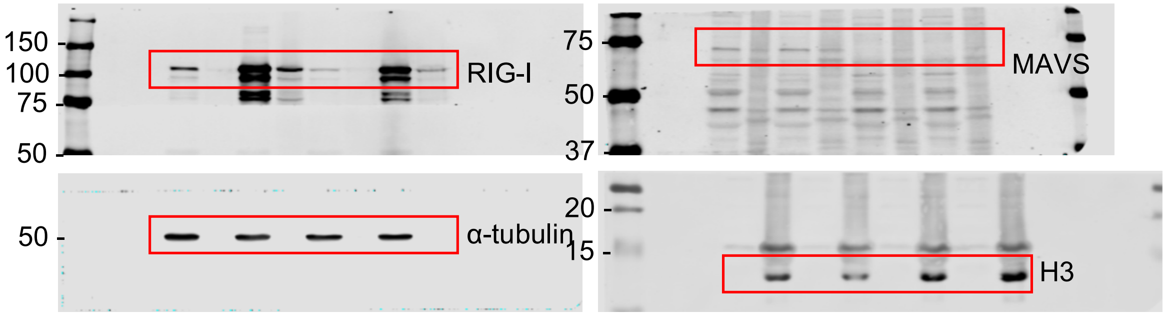

Figure 2d

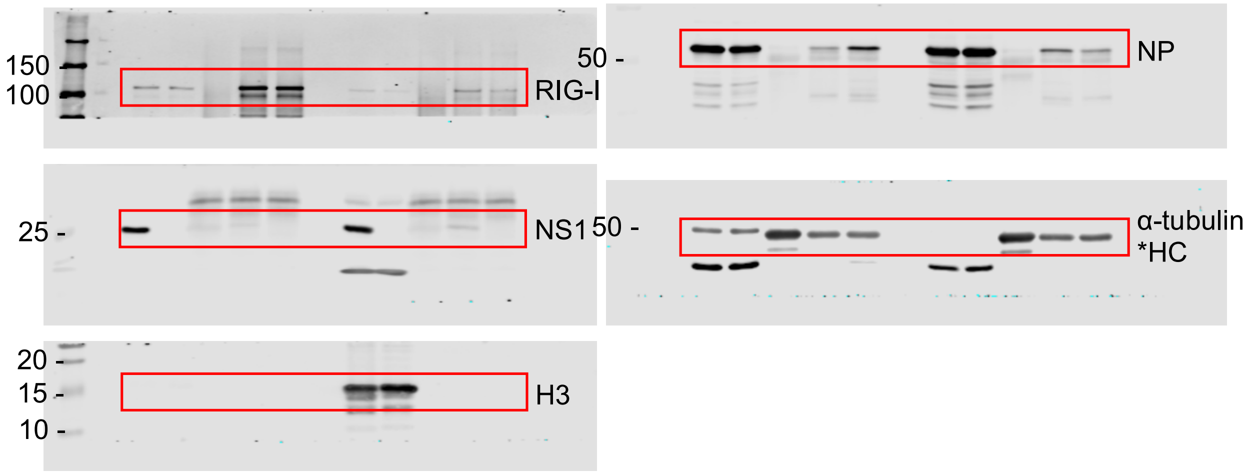

Figure 2e

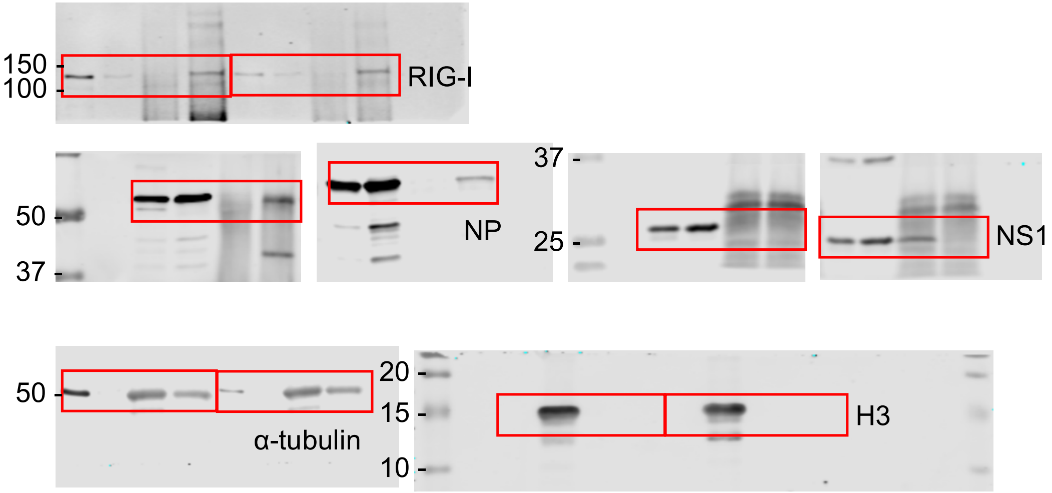

Figure 2f

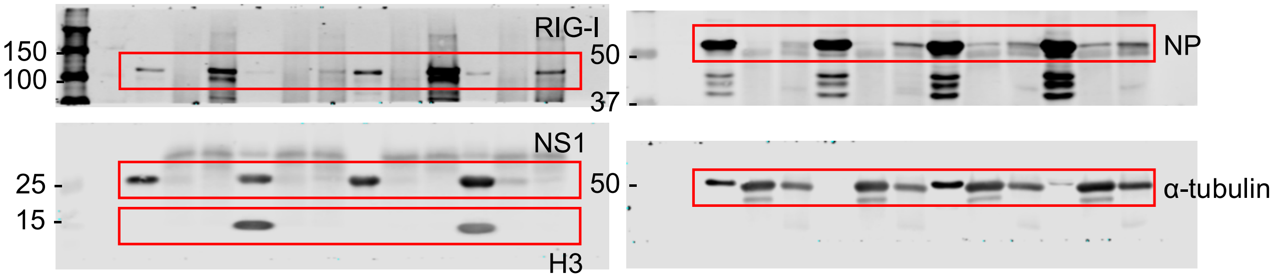

Figure 3a

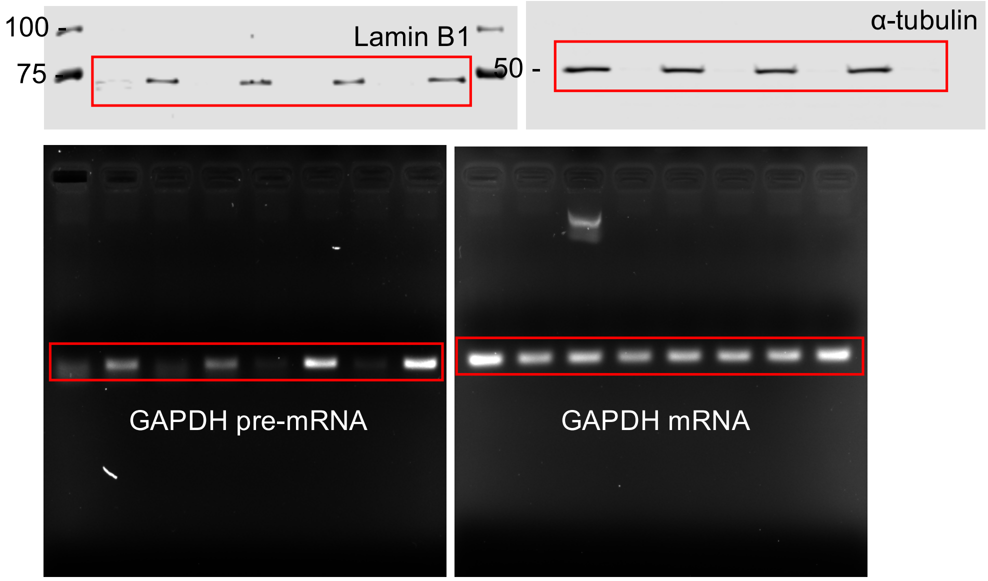

Figure 3c

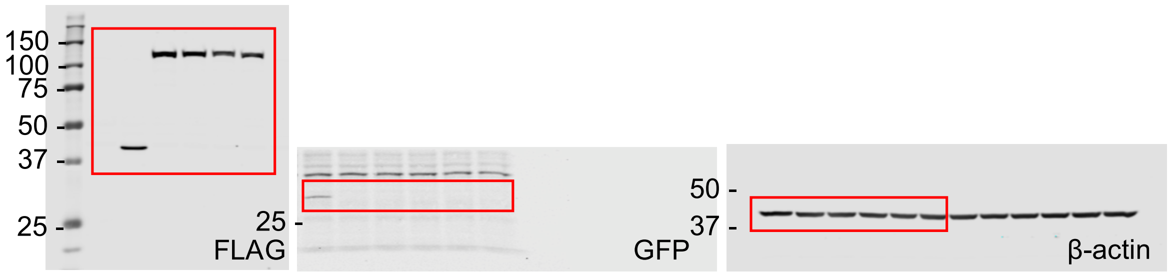

Figure 4b

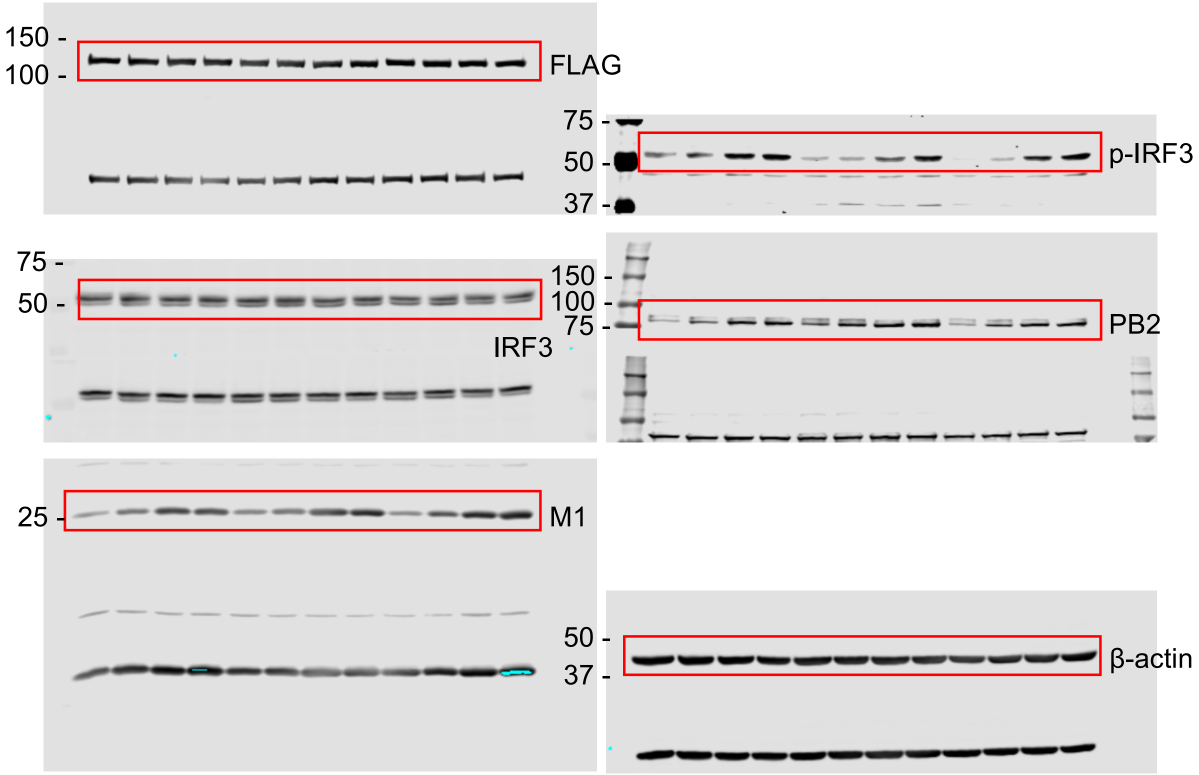

Figure 4d

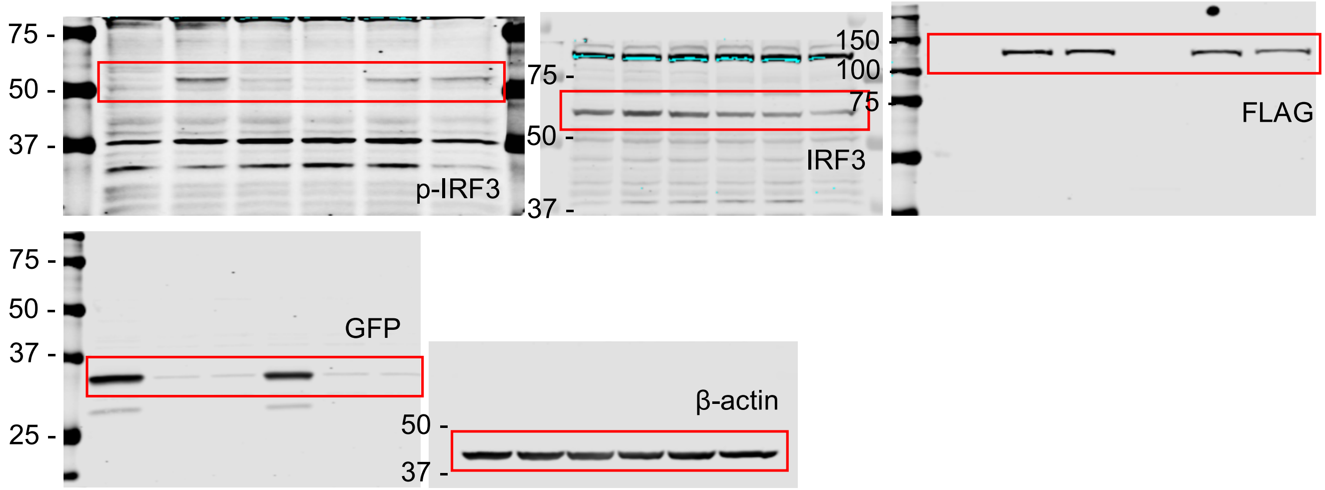

Figure 4f

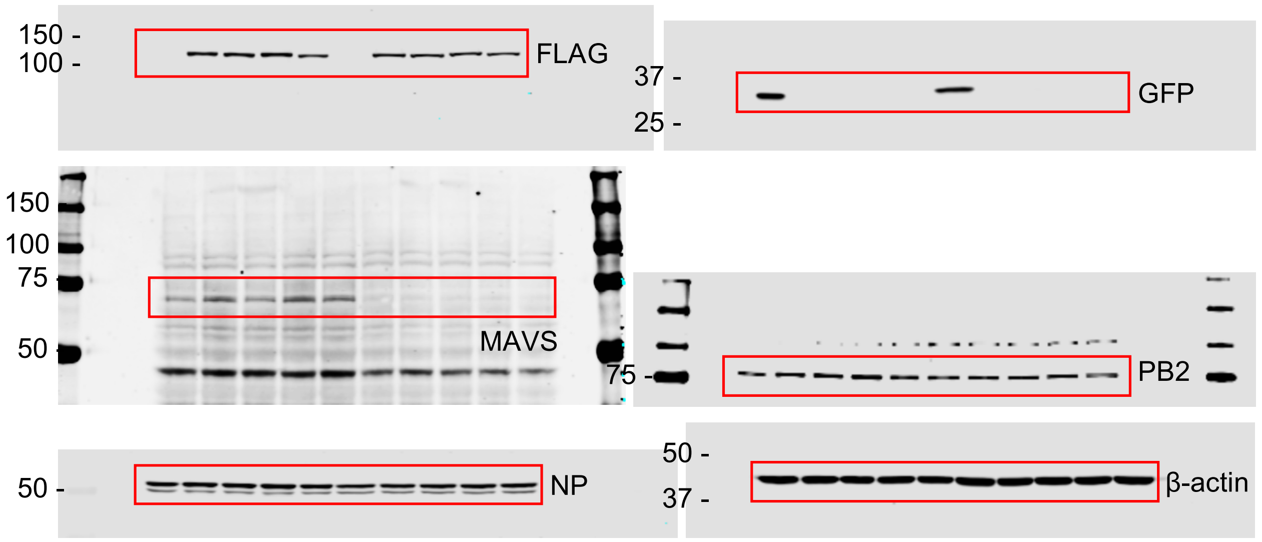

Figure 4g

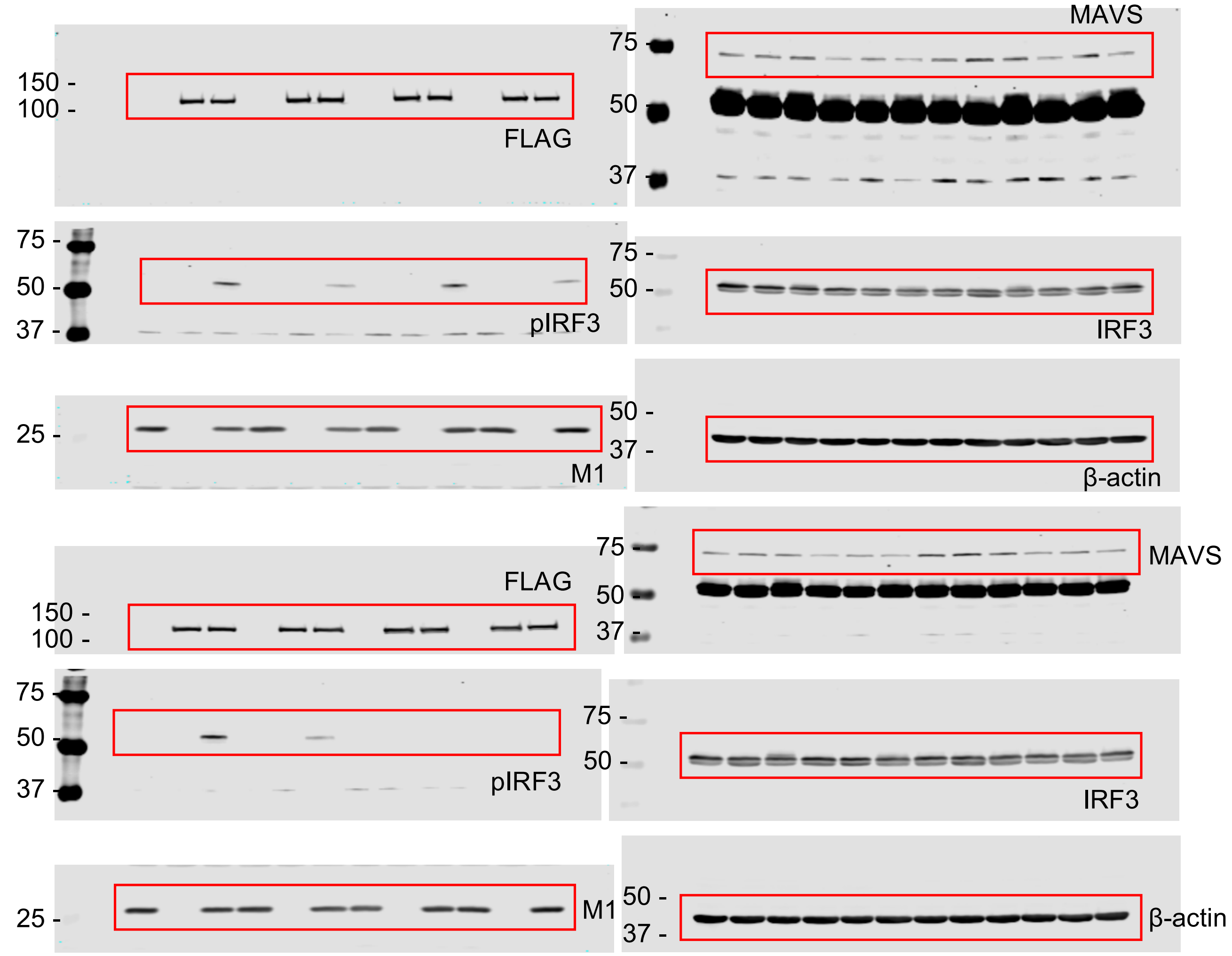

Figure 5c

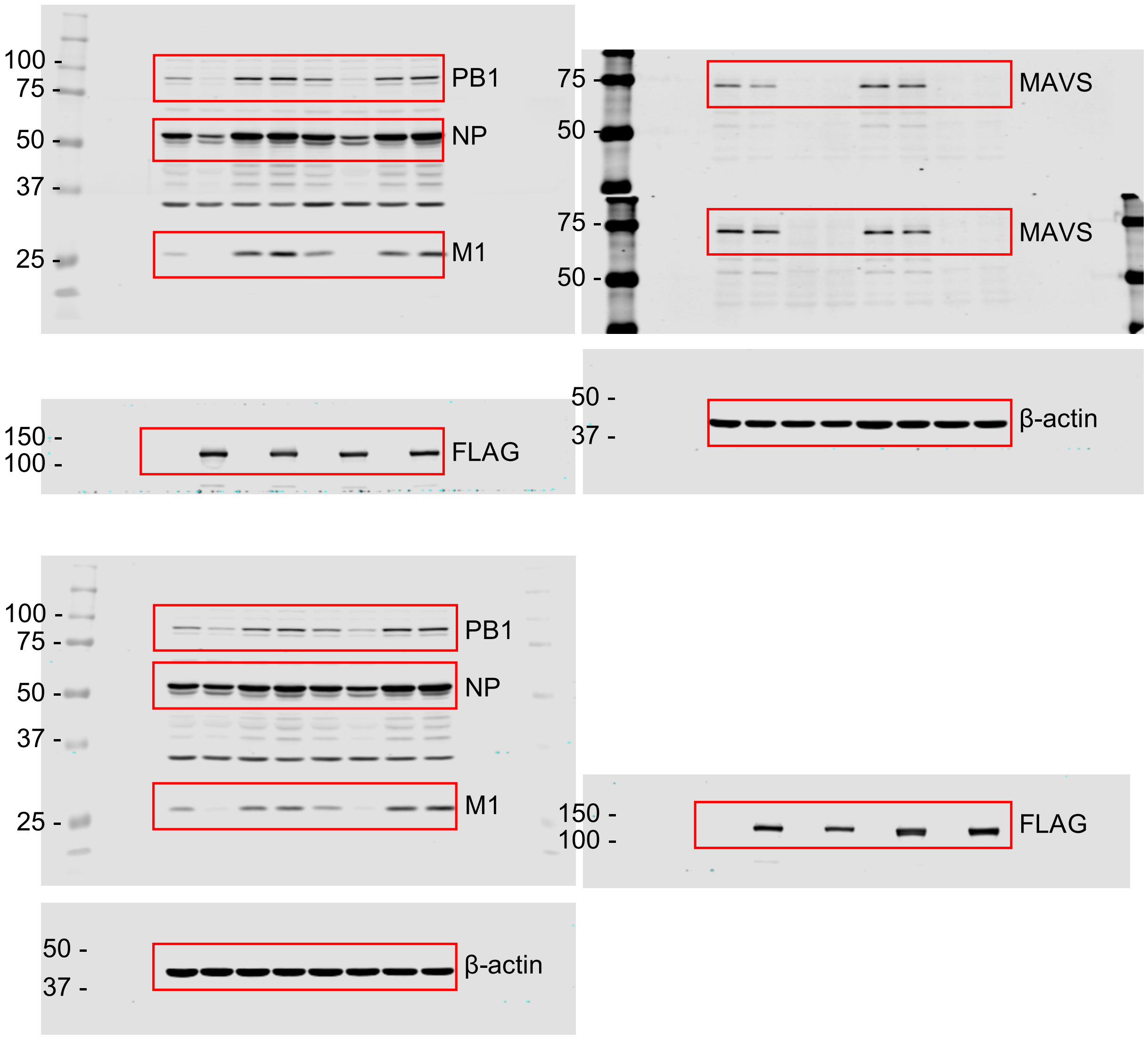

Figure 6c

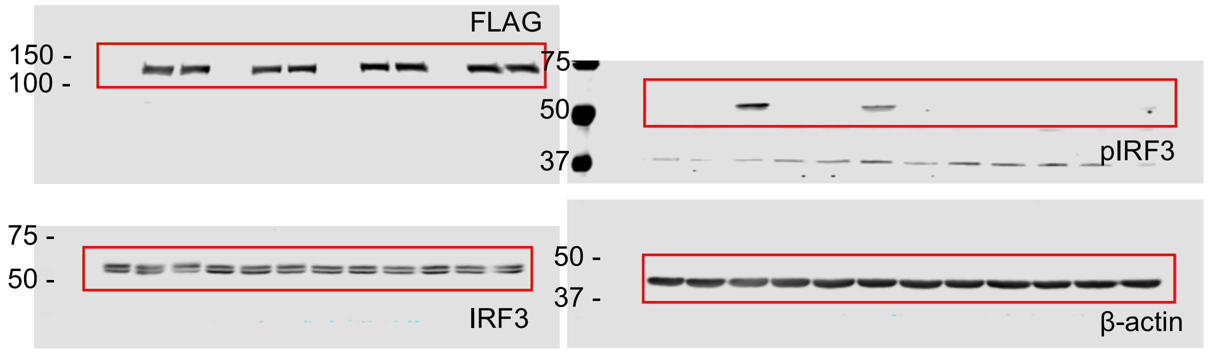

Figure 6d

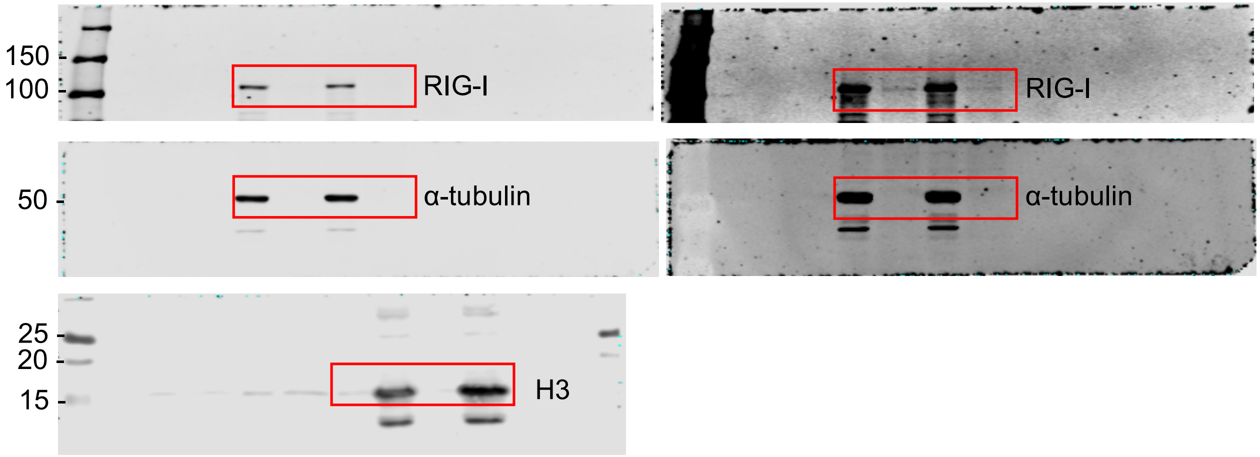

Suppl. Figure 1c

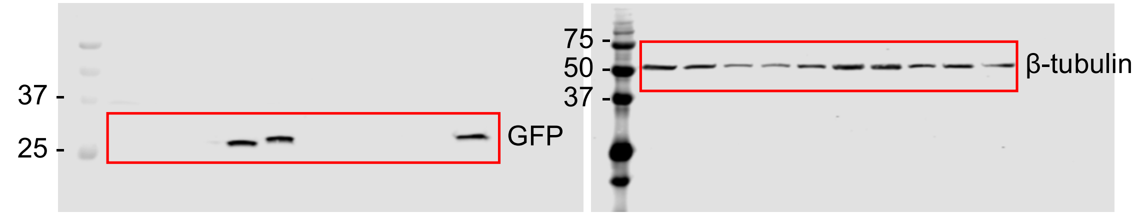

Suppl. Figure 1d

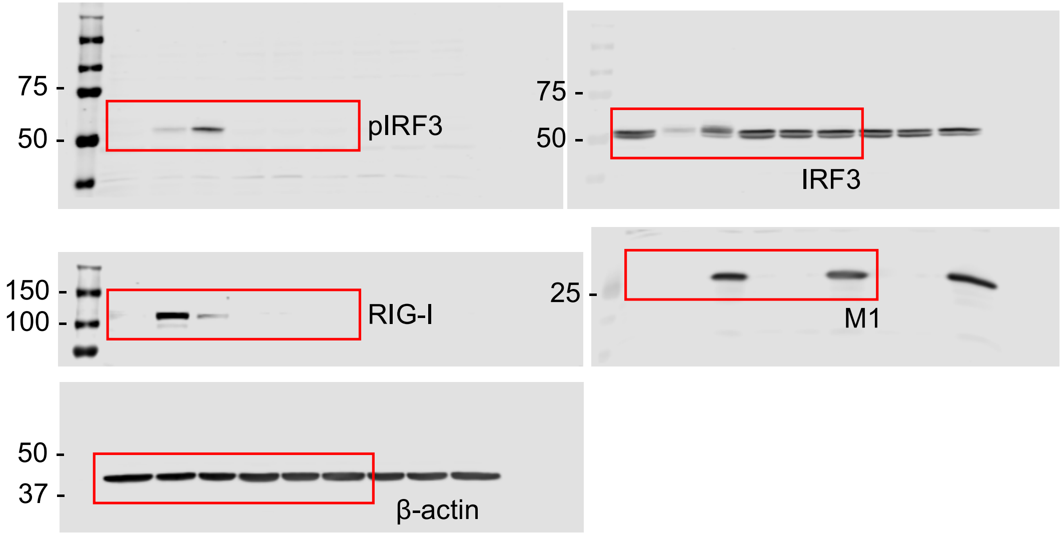

Suppl. Figure 2e

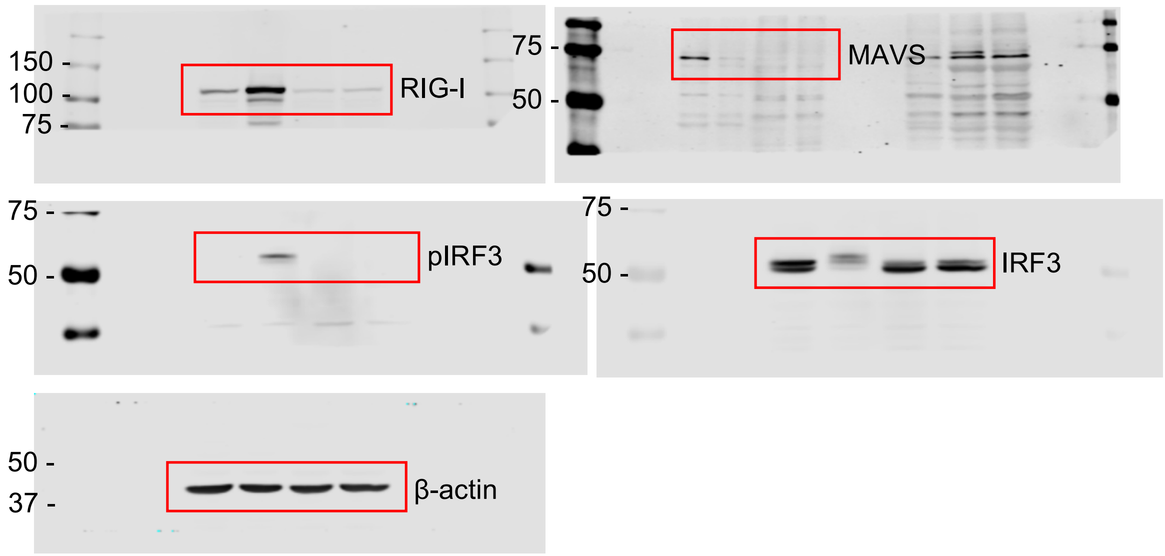

Suppl. Figure 4a

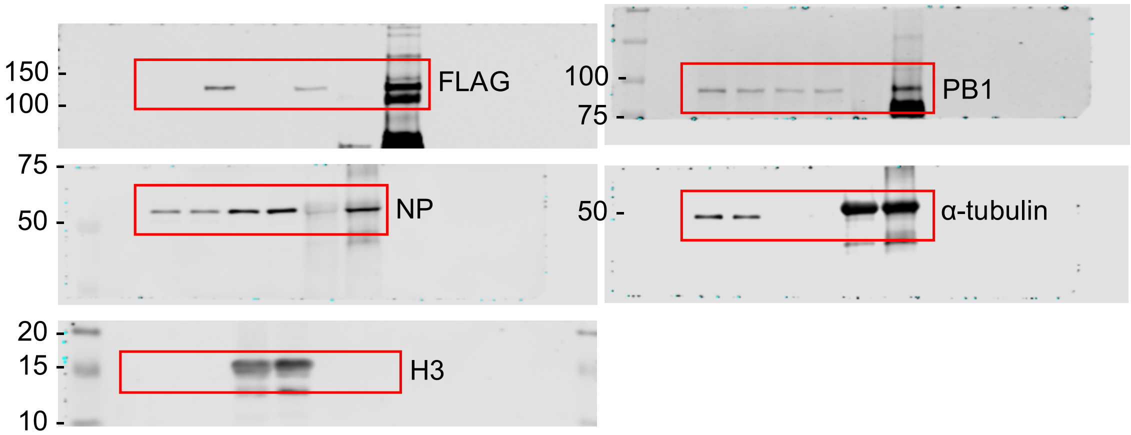

Suppl. Figure 5b

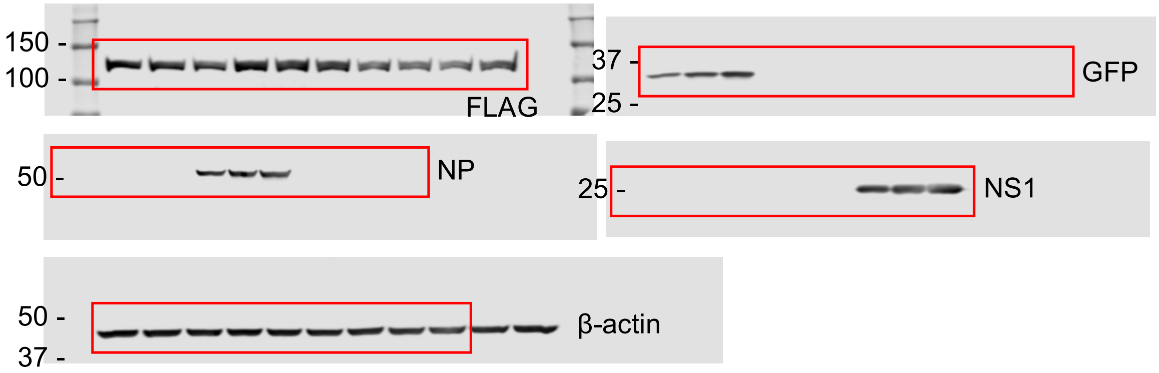

Suppl. Figure 5c

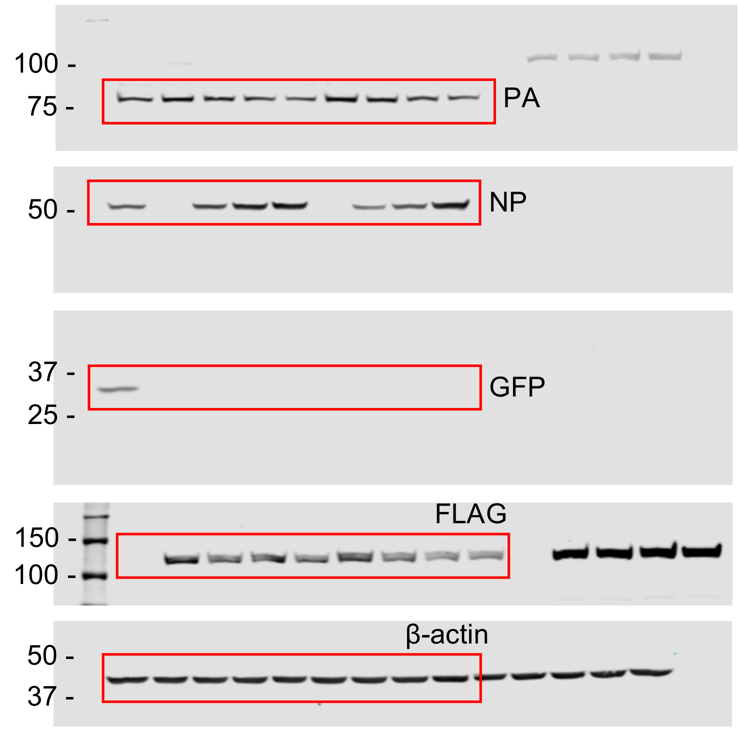

Suppl. Figure 6a

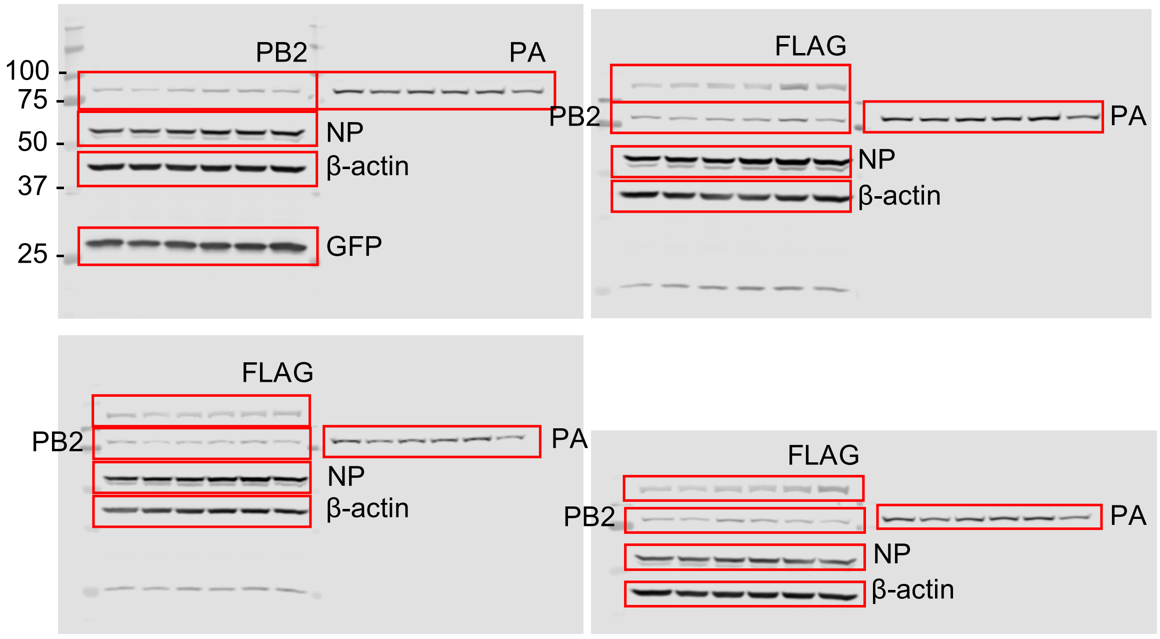

Suppl. Figure 7

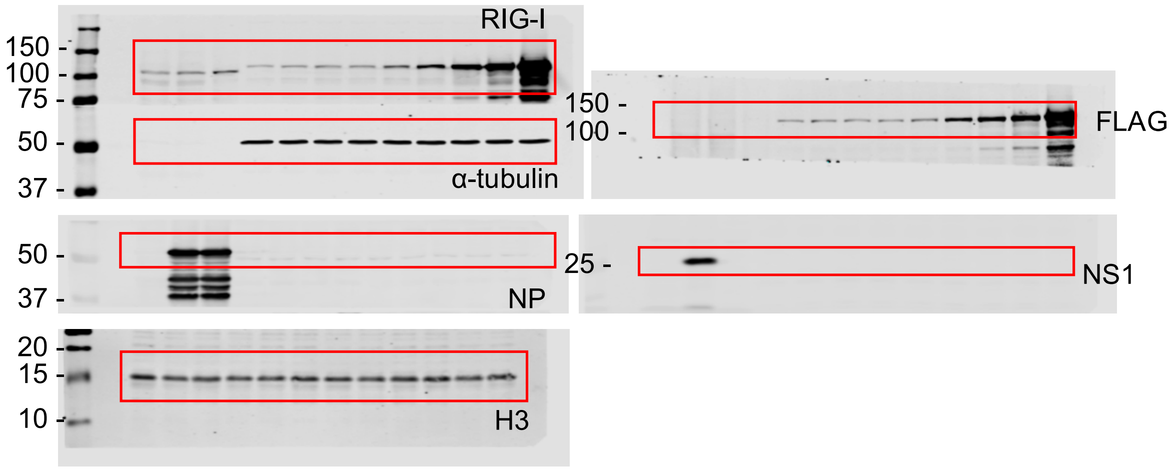

Suppl. Figure 8

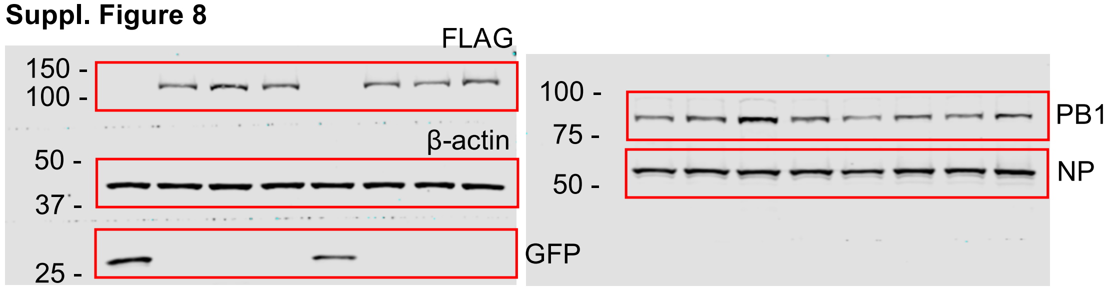

Suppl. Figure 9a

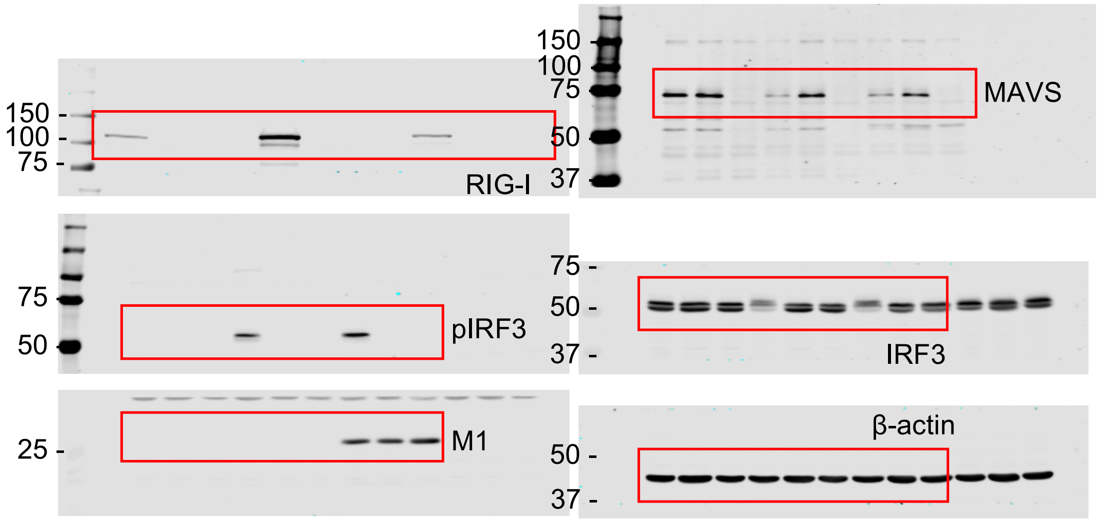

Supplementary Table 1. Cloning and sequencing primers used in this study.

Site-directed mutagenesis		
**Construct**	**Forward (5' to 3')**	**Reverse (5' to 3')**
PB1-D445A/D446A	GGGATGGTCTTCAATCCTCTGCCGCTTTTGCTCTGATTGTGAATGCAC	GTGCATTCACAATCAGAGCAAAAGCGGCAGAGGATTGAAGACCATCCC
PB2-R142A	CCATTTTAGAAACCAAGTCAAAATAGCTCGGAGAGTTGACATAAATCCT	AGGATTTATGTCAACTCTCCGAGCTATTTTGACTTGGTTTCTAAAATGG
PB2-E361A	ATAAGAGTGCATGAGGGATATGCAGAGTTCACAATGGTTGGGAG	CTCCCAACCATTGTGAACTCTGCATATCCCTCATGCACTCTTAT
PA-D108A	GAGAAACCAAAGTTTCTACCAGCTTTGTATGATTACAAGGAGAATAG	CTATTCTCCTTGTAATCATACAAAGCTGGTAGAAACTTTGGTTTCTC
PA-E410A	CTTGCAAGTTGGATTCAGAATGCGTTTAACAAGGCATGCGAACTG	CAGTTCGCATGCCTTGTTAAACGCATTCTGAATCCAACTTGCAAG
RIG-I-T55I	GAGGCTGCCATACTTTTTCTC	CATTGGGCCCTTGTTGTTTTTC
RIG-I-K270A	AGGTTGTGGAGCAACCTTTGTTTC	GTAGGAGCACATATTATTGTGTTTTTTCC
RIG-I-K888E	TCCAGTTATAgAAATTGAAAGTTTTGTG	ATCTCAAATGTCTTGTACTTC
**Compartment-specific RIG-I**		
RIG-I-SLN	TCTTAGGGGAGGAGGTATGACCACCGAGCAGCGACG	AAAAGAGGAAGGTTCGCGGCCGCAAGCTTGTC
RIG-I-NES	CACACTGTACCTCGGAGGAGGTATGACCACCGAGCAGCGACG	CTCAGCTGCTTCTTGAGCGCGGCCGCAAGCTTGTC
RIG-I-NLS	GAAGGTTGGAGGAGGTATGACCACCGAGCAGCGACG	CTCTTTTTCTTAGGCGCGGCCGCAAGCTTGTC
**CRISPR sequencing**		
U6	GAGGGCCTATTTCCCATGATTCC	
RIG-I Indel	CCGCCGCTAGTTGCACTTTC	CCCAGAAGCCTCTGCTCATCTC
**DI detection**		
PR8-NA	AGCAAAAGCAGGAGTTTAAAATGAATCC	AGTAGAAACAAGGAGTTTTTGAACAGAC

Supplementary Table 2. qRT-PCR primers used in this study.

Strand-specific qRT-PCR		
**RT primers**	**Sequences (5' to 3')**	
PR8 NA-vRNA RT	GGCCGTCATGGTGGCGAATCTATAATGACTGATGGCCCGAGT	
PR8 NA-cRNA RT	GCTAGCTTCAGCTAGGCATCAGTAGAAACAAGGAGTTTTTTGAAC	
PR8 NA-mRNA RT	CCAGATCGTTCGAGTCGTTTTTTTTTTTTTTTTTTGAACAGACTAC	
**PCR primers**	**Forward (5' to 3')**	**Reverse (5' to 3')**
PR8 NA-vRNA	GGCCGTCATGGTGGCGAAT	TTCGAACCATGCCAATTGTC
PR8 NA-cRNA	CTGTATGAGGCCGTGCTTCTG	GCTAGCTTCAGCTAGGCATC
PR8 NA-mRNA	CTGTATGAGGCCGTGCTTCTG	CCAGATCGTTCGAGTCGT
**qRT-PCR**		
**Target gene**	**Forward (5' to 3')**	**Reverse (5' to 3')**
GAPDH	TGCACCACCAACTGCTTAGC	GGCATGGACTGTGGTCATGAG
Pre-GAPDH	CTGGCTTTCCCATAATTTCC	CCAGTAGAGGCAGGGATGAT
GUSB	GGTGCTGAGGATTGGCAGTG	CGCACTTCCAACTTGAACAGG
IFNB	AAACTCATGAGCAGTCTGCA	AGGAGATCTTCAGTTTCGGAGG
IFNL1	CGCCTTGGAAGAGTCACTCA	GAAGCCTCAGGTCCCAATTC
IP10	CCTGCTTCAAATATTTCCCT	CCTTCCTGTATGTGTTTGGA
IAV NP	GTCTTCGAGCTCTCGGAC	CCTCTGCATTGTCTCCGAA
SeV N	GCTGAACGGTTAGAGGAGGAAAC	CGTGATCGACACCGTTATTGC
HBV pgRNA	TGTTCAAGCCTCCAAGCT	GGAAAGAAGTCAGAAGGCAA
